# Molecular and pathobiological insights of bikunin/UTI in cancer

**DOI:** 10.1007/s11033-022-08117-2

**Published:** 2022-11-21

**Authors:** Antonio Junior Lepedda, Gabriele Nieddu, Claudia Cannas, Marilena Formato

**Affiliations:** Department of Biomedical Sciences, University of Sassari, Sassari, Italy

**Keywords:** Bikunin, Urinary Trypsin Inhibitor, Ulinastatin, Cancer invasiveness, uPA/uPAR system, High-throughput proteogenomics, Biomarker discovery

## Abstract

Bikunin is a small chondroitin sulfate proteoglycan (PG) with Ser-protease inhibitory activity that plays pleiotropic roles in health and disease. It is involved in several physiological processes including stabilization of the extracellular matrix (ECM) of connective tissues and key reproductive events. Bikunin is also implicated in both acute and chronic inflammatory conditions and represents a non-invasive circulating and/or urinary (as Urinary Trypsin Inhibitor or UTI) biomarker. It exerts inhibitory effects on urokinase-type plasminogen activator (uPA) and its receptor (uPAR) mediating tumor invasiveness by a down-regulation of uPA mRNA expression, thus representing an anti-metastatic agent. However, only limited data on its potential as a diagnostic and/or prognostic marker of cancer have been reported so far. Recent technological advances in mass spectrometry-based proteomics have provided researchers with a huge amount of information allowing for large-scale surveys of the cancer proteome. To address such issues, we analyzed bikunin expression data across several types of tumors, by using UALCAN proteogenomic analysis portal. In this article we critically review the roles of bikunin in human pathobiology, with a special focus on its inhibitory effects and mechanisms in cancer aggressiveness as well as its significance as cancer circulating biomarker.

**Supplementary Information:**

The online version contains supplementary material available at 10.1007/s11033-022-08117-2.

## Introduction

Cancer is an ever-growing healthcare problem, being the second leading cause of death globally, with an estimated 9.6 million deaths in 2018 (https://www.who.int/health-topics/cancer). It is a group of related diseases caused by genetic mutations leading to abnormal cell proliferation and spreading throughout the body.

Tumor invasion is a hallmark of human cancer and is the major contributor to the morbidity and mortality of the disease. Although many signs of progress have been done in developing therapeutic strategies against primary tumors, targeting tumor metastasis has achieved only limited success. This process involves a deep remodeling of the pre-existing ECM, which physiologically provides cells with chemical signals and mechanical support essential for maintaining tissue homeostasis [1], allowing to support the continued expansion of the tumor mass and the metastatic process [2]. Among the several matrix-degrading enzymes implicated in this process there are proteases such as matrix metalloproteinases (MMPs), plasminogen activators, cathepsins, as well as glycosidases such as heparanase and hyaluronidases [3]. In this respect, a large body of evidence shows that the urokinase-type plasminogen activator and its receptor (uPA/uPAR system) play key roles in mediating proteolysis during cancer invasion and metastasis and correlate with both tumor invasiveness and poor prognosis [4]. Indeed, the serine protease uPA converts plasminogen into plasmin, another serine protease that, in turn, may degrade several components of both the basement membrane and the ECM, including laminin, vitronectin, and fibronectin, therefore, facilitating the migration of tumor cells through the ECM and basement membrane barriers. Expression of uPA and uPAR can be upregulated by mitogens, growth factors, oncogenes, and binding of integrin with ECM proteins [5]. Hence, the uPA/uPAR system may represent a potentially relevant therapeutic target [4].

Bikunin is a small circulating proteoglycan (PG) found also in urine, as Urinary Trypsin Inhibitor (UTI) or ulinastatin, and in amniotic fluid, with inhibitory activity against serine proteases. Although bikunin cannot inhibit directly uPA activity, it exerts a down-regulation of uPA mRNA expression and protein secretion leading to an inhibition of both in vitro and in vivo tumor cell invasion and metastasis [6–9]. In this article, we critically review the roles of bikunin in human pathobiology, with a particular emphasis on cancer propagation. Furthermore, its significance as a potential diagnostic and/or prognostic biomarker in various cancer types will be discussed, also in light of the new information obtained by analyzing the most recent cancer proteogenomics databases.

## Search strategy and data analysis

A comprehensive literature search of the Medline/PubMed database was performed on the following topics: bikunin/urinary trypsin inhibitor structure and metabolism, roles in different pathophysiological conditions with a particular emphasis on tumors cells invasion through the uPA/uPAR system, usefulness as tissue and circulating biomarker. This manuscript reviews the major literature on this research field since 1995 and shows an overview of the main findings. Furthermore, it provides insight into the potential of bikunin as a cancer biomarker by analyzing the most recent high-throughput gene- and protein-expression databases, performed with the search engine UALCAN (The **U**niversity of **AL**abama at Birmingham **CAN**cer data analysis Portal). UALCAN is an interactive web resource (http://ualcan.path.uab.edu/index.html) that allows for the analysis of publicly available cancer OMICS data. Among the possible applications, it enables the identification of biomarkers or the in silico validation of potential genes of interest. Furthermore, it provides graphs and plots depicting expression profile and patient’s survival information. The available databases have been provided by The Clinical Proteomic Tumor Analysis Consortium (CPTAC) (https://gdc.cancer.gov/about-gdc/contributed-genomic-data-cancer-research/clinical-proteomic-tumor-analysis-consortium-cptac), and by The Cancer Genome Atlas (TCGA) consortium (https://www.cancer.gov/about-nci/organization/ccg/research/structural-genomics/tcga). The CPTAC of the National Cancer Institute (NCI) at the National Institutes of Health (NIH) is devoted to accelerating the understanding of the molecular basis of cancer through the application of large-scale proteome and genome analyses, or proteogenomics. TCGA is a landmark cancer genomics program developed by the joint effort between NCI and the National Human Genome Research Institute.

## Bikunin/UTI structure and biosynthesis

**Fig. 1:**
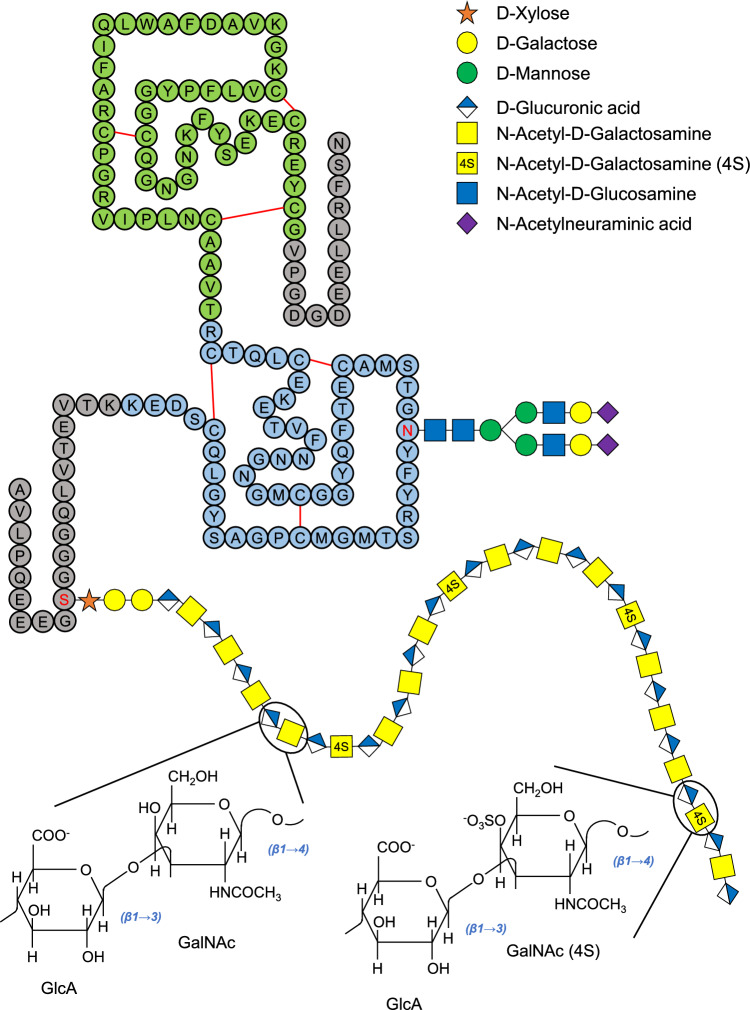
Representative schematic structure of bikunin/UTI. Amino acid residues are reported as circles, while monosaccharides are drawn according to the Symbol Nomenclature for Glycans (https://www.ncbi.nlm.nih.gov/glycans/snfg.html). Kunitz I and II domains are reported in light blue and green, respectively. The low-sulfated CS chain, composed of 12–18 repeating disaccharide units consisting of glucuronic acid (GlcA) and N-acetyl galactosamine (GalNAc), is bound to Ser^10^, while a biantennary N-linked oligosaccharide is bound to Ans^45^. Chemical structures of the CS non-sulfated (left) and monosulfated at C-4 (right) disaccharide units are reported

Bikunin is a chondroitin sulfate PG (CSPG) with multipotent inhibitory effect on several Ser proteinases, including trypsin, chymotrypsin, granulocyte elastase, kallikrein, cathepsin G, acrosin, and plasmin [10]. It carries both an O-linked low-charge CS chain, at Ser^10^, and a N-linked oligosaccharide, at Asn^45^ [10–12]. Its protein moiety consists of 147 amino acid residues folded in two Kunitz-type domains (from residues 22 to 77 and from 78 to 133, respectively), containing three disulfide bonds each, as well as N- and C-terminal sequences of 10–25 amino acid residues [11] (Fig. 1).

The whole CSPG has a molecular mass of about 25–26 kDa, being composed of a 16 kDa polypeptide chain, a 2 kDa N-linked oligosaccharide and a 7 kDa CS chain. This latter is constituted of 12–18 disaccharide repeating units, consisting of glucuronic acid (GlcA) and N-acetyl galactosamine (GalNAc), connected to Ser^10^ via a linkage tetrasaccharide (Xyl-Gal-Gal-GlcA). About 25% of GalNAc residues are sulfated at C-4, frequently near the reducing end of CS chain [13–15] (Fig. 1).

Bikunin is synthesized primarily by hepatocytes as a precursor of 352 amino acids (AMBP protein), containing also alpha-1-microglobulin, encoded by Alpha-1-Microglobulin/Bikunin Precursor (AMBP) gene (located on chromosome 9q32), which consists of 10 exons and 9 introns of about 18 kb length, overall (https://www.ncbi.nlm.nih.gov/gene/259) [16]. During maturation, the precursor is proteolytically cleaved into alpha-1-microglobulin (residues from 1 to 205) and bikunin (residues from 206 to 352) [17]. Following glycosidic modification in the Golgi apparatus, bikunin is covalently linked to the C-terminal amino acid residue of one or two polypeptides, called the heavy chains (HCs), through an ester bond with a non-sulfated GalNAc residue of the CS chain, and is released in plasma almost exclusively as a subunit of inter alpha inhibitor (IαI) family molecules [18–20].

In addition to liver parenchyma, which is the main site of AMBP protein synthesis, this gene is expressed at low levels in many tissues/organs (https://gtexportal.org/home/gene/ENSG00000106927#eQTLBlock; https://www.proteomicsdb.org/proteomicsdb/#protein/proteinDetails/51583/expression). For its part, bikunin is widely detectable due to both in situ synthesis and adsorption from circulation [21]. Bikunin expression in tissues varies widely also in relation to pathophysiological conditions including cancer, as shown in the last part of this review.

## Pathobiological roles of bikunin/UTI

IαI molecules are recruited from circulation to extravascular sites, where the HCs are transferred from the CS chain of bikunin to the locally synthesized hyaluronan (HA), thus forming the serum-derived HA-associated protein-HA complex (SHAP-HA), which supports the formation of new ECM and its stabilization. The formation of this complex has been often associated with inflammatory conditions [19]. Bikunin is essential in these processes because it is required for presenting HCs to HA, as proven by gene knock out studies carried out on murine model [22].

The transfer of HCs from the CS chain of bikunin to the C-6 of the GlcNAc residues of cell-secreted HA, by a transesterification reaction to form the SHAP-HA complex, requires the formation of a covalent intermediate between HC and the transferase tumor necrosis factor stimulated gene-6 protein (TSG-6), followed by the release of bikunin [23–25]. In this process, the bikunin CS chain sulfation pattern has been reported to be crucial for the transesterification of HCs to HA [26].

The release of bikunin from IαI molecules is considered a regulatory mechanism since it leads to the recovery of some bikunin activities that are lost when the complex is formed. In this context, besides its Ser-proteinase inhibitory activities [27, 28], bikunin is involved in inhibition of lipopolysaccharide-induced lethality [29–31], in smooth muscle contraction [32, 33], in neutrophil release of elastase [34], in mast cell release of histamine [35], in suppression of immune cells [36, 37], and in urolithiasis [38, 39], as well as in stabilization of lysosomal membranes [40, 41], representing a substantial anti-inflammatory agent [42]. Recent studies have shown that bikunin can also play important anti-fibrotic and organ protective roles with important therapeutic implications in chronic kidney disease [43].

Bikunin plays also key roles in different reproductive events, such as cumulus oocyte complex formation, pregnancy, and delivery, beyond being related to the most common pregnancy complications such as pre-term delivery, pre-eclampsia, and gestational diabetes, of which it may represent a non-invasive marker [44]. Because of its proteinase inhibitory activity, bikunin is quickly removed from circulation (approximately 7 min) to prevent a shutdown of repair and healing processes, by both tissue uptake and renal excretion and it is found in urine as UTI or ulinastatin [14]. In human physiological conditions, UTI levels are lower than 5 μg/mL, but they may rise up to tenfold as a consequence of both acute and chronic inflammations including bacterial or viral infections, liver diseases, cancer, type I and II diabetes mellitus, and renal diseases [45–53]. Similarly, modifications of both sulfation degree and chain length of CS moiety have been reported following inflammatory conditions [54]. Hence, bikunin can be considered a positive acute phase protein [42, 54–56].

Bikunin determination is mainly performed by enzyme inhibition assays or immunological detection. The formers allow for a rapid evaluation of the biological activity of bikunin as trypsin inhibitor, based on color shift, with limited specificity and sensitivity. On the other hand, ELISA, immunohistochemistry, and western blotting analyses are all affected by the specificity of the antibody reaction. Furthermore, ELISA cannot discriminate between bikunin/UTI and IαI, whereas, immunohistochemistry and western blotting, due to their intrinsic characteristics, do not allow absolute and reproducible protein quantitation [52].

## Bikunin/UTI roles in tumors cell invasiveness

In this section we summarize and discuss on the current knowledge on the inhibitory effects and mechanisms of this small CSPG in cancer invasiveness, using “bikunin” or “UTI”, according to the authors that described them.

UTI exerts its inhibitory effects on cell motility and invasiveness by forming membrane complexes with UTI-binding proteins, leading to down-regulation of uPA mRNA expression through negative regulation of PKC- and MEK1/ERK2/c-Jun-dependent signaling pathways [57].

At least two binding sites specific for UTI have been identified on the surface of both neoplastic (human choriocarcinoma SMT-cc1, human chondrosarcoma HCS-2/8, human promyeloid leukemia U937, and murine Lewis lung carcinoma 3LL cells) and normal (human neutrophils, human umbilical vein endothelial cells, fibroblasts, and myometrial cells) cells, a 40-kDa UTI-Binding Protein (UTI-BP40) and a 45-kDa UTI-BP (UTI-BP45) [58]. UTI-BP40 is very similar to a truncated form of human cartilage link protein (LP) and is attached to the cell membrane via a hyaluronic acid anchor [59], whereas UTI-BP45 is a putative CD44 accessory protein [58]. By using truncated forms of UTI (non-glycosylated UTI or carboxyterminal fragments), Hirashima et al. demonstrated that the binding of UTI to UTI-BP45 occurs via the CS moiety [58]. Furthermore, they found that incubation of human chondrosarcoma cell line HCS-2/8 with an anti-LP antibody or soluble chondroitin 4-sulfate, by blocking the binding to either UTI-BP40 or UTI-BP45, independently abrogated suppression by UTI of phorbol ester-induced uPA expression, therefore demonstrating that the concurrent binding to both BPs by UTI is necessary to inhibit uPA expression.

A more in-depth analysis of the mechanisms which regulate UTI binding to cells was performed by Suzuki et al. using UTI derivatives such as the O-glycoside-linked N-terminal glycopeptide, the N-glycoside-linked C-terminal tandem Kunitz domains, CS devoid UTI, asialo UTI, UTI lacking the N-linked oligosaccharide, and the Kunitz domain II of UTI [60]. By this approach, they found that the presence of either the N-terminal moiety (Ala^1^ to Lys^21^ residues) containing the chondroitin 4-sulfate side chain or the Kunitz domain I (Lys^22^ to Arg^77^ residues) was necessary to bind cells, whereas the uPA expression was inhibited only by the UTI derivative lacking the N-linked oligosaccharide [60]. Further studies showed that the binding of UTI to its receptor reduces uPA expression by hindering CD44 clustering (activation) and interaction with HA [61].

In ovarian cancer cells, UTI was found effective in inhibiting uPA expression induced by TGF-β1-mediated calcium release, which is a common signaling response in malignant and non-malignant cells treated with a variety of growth factors [7], as well as in inhibiting plasminogen activator inhibitor type-1 (PAI-1) and collagen synthesis through inhibition of TGF-β1 receptor clusterization [62]. Further confirmation of the described bikunin-mediated suppression of cell invasiveness came from transfection studies in which ovarian cancer cells overexpressing bikunin exhibited significantly reduced uPA mRNA levels, inhibition of ERK1/2 phosphorylation, and an overall reduced invasion capacity, whereas proliferation, adhesion, or migration were unchanged [63].

To identify the whole panel of bikunin-regulated genes, high throughput cDNA microarray screening against 3,126 sequence-verified clones was performed on bikunin-treated or bikunin-transfected ovarian cancer cell lines. The 11 bikunin-stimulated genes and 29 bikunin-repressed genes included transcriptional regulators, oncogenes/tumor suppressor genes, signaling molecules, growth/cell cycle, invasion/metastasis, cytokines, apoptosis, ion channels, ECM proteins, as well as some proteases, according to the recognized anti-metastatic effects of bikunin [63].

Studies on breast cancer showed that UTI may inhibit MCF-7 cell line proliferation and the growth of xenograft breast cancer in nude mice by reducing the expression of the receptor 4 for the CXC chemokine and MMP-9 [8]. Furthermore, UTI was shown to inhibit invasion and metastasis in both primary and MDA-MB-231 cells and in a xenograft mouse model [9]. Inhibitory effects on cell proliferation, cell invasion, and migration by UTI administration were also shown in gastric cancer SGC-7901 cells, where a downregulation of uPA expression and activity were evidenced [64]. The in vitro evidence linking UTI to uPA expression and inhibition of tumor cells invasiveness was also corroborated by an immunohistochemical study on 89 ovarian cancer specimens showing a negative correlation between tissue bikunin and uPA levels [65]. Further support for in vitro findings came from bikunin knockout mice that showed an increased prevalence of lung metastasis with respect to Bik^+/+^ mice, which was significantly reduced by administration of exogenous bikunin [66]. The therapeutic potential of bikunin was also evaluated in a phase I trial involving nine patients with advanced uterine cervical carcinoma after definitive treatment, showing that bikunin was well tolerated by oral administration and was effective in suppressing uPA expression in tumor [67].

## Significance of bikunin/UTI as tissue and circulating biomarker of cancer

Beside the plethora of in vitro mechanistic studies dealing with the inhibitory pathways of tumor invasiveness mediated by bikunin, only scanty data on its potential as diagnostic and/or prognostic marker of cancer have been reported so far. Among them, the above-mentioned immunohistochemical work of Tanaka and co-workers [65] showed bikunin as an independent predictor for disease-free survival and overall survival in ovarian cancer. They performed a retrospective study on the expression of bikunin, uPA and CD68 (macrophages) in 89 tumor specimens, evidencing that i) bikunin localized mainly in the cytoplasm of tumor-infiltrating macrophages, ii) its levels were inversely related with those of uPA, iii) low expression of bikunin was inversely related with lymph node metastasis and disseminated peritoneal metastasis. The 49 patients with low bikunin expression had a 5-year survival rate of 39% compared to the 63% obtained for the 40 patients with high bikunin levels. They concluded the study suggesting that macrophages-derived bikunin might represent an antiinvasive factor, most likely acting through a down-regulation of tumor-associated uPA expression, with prognostic value [65]. Also, bikunin plasma concentrations were found positively associated with improved survival and patient outcomes [47, 68]. In this respect, Matsuzaki et al. determined bikunin levels in plasma samples, by using ELISA, from 200 healthy controls, 200 patients with benign gynecologic diseases, and 327 patients with ovarian cancer, after treatment with chondroitin lyase ABC. Although the enzymatic pre-treatment did not allow to discriminate between the complex and the free form, and any tumor-specific bikunin isoform was evidenced, they were able to identify plasma bikunin as a strong and independent favorable prognostic marker for ovarian cancer.

Recently, Sekikawa et al., by immunohistochemical analysis on 95 oral squamous cell carcinoma specimens, observed the underexpression of AMBP precursor (using a monoclonal antibody raised against a full-length recombinant AMBP) significantly associated with a high metastatic potential to cervical lymph nodes and a poor overall survival [69].

**Fig. 2:**
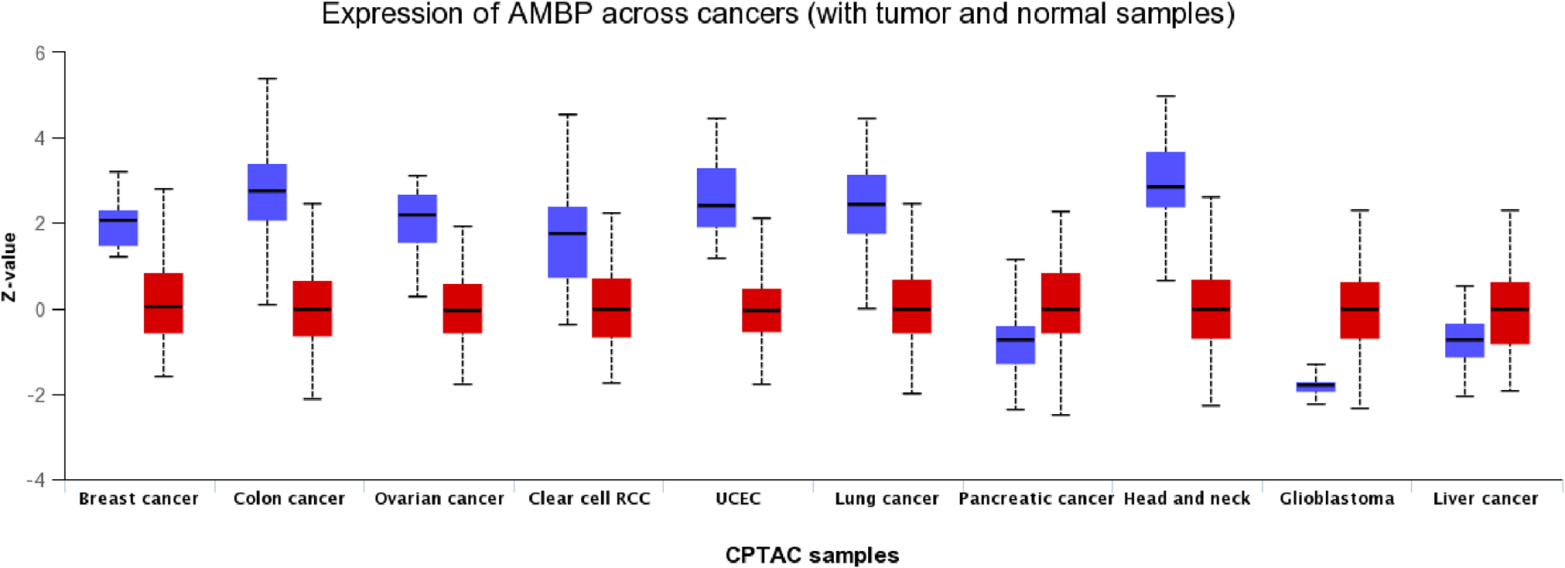
Box-Whisker plot showing AMBP proteomic expression profile across cancers (blue, normal; red, tumor) obtained by using the web resource UALCAN (http://ualcan.path.uab.edu/cgi-bin/Pan-cancer-CPTAC.pl?genenam=AMBP). UALCAN provides protein expression analysis option using data from Clinical Proteomic Tumor Analysis Consortium (CPTAC) and the International Cancer Proteogenome Consortium (ICPC) datasets [70, 71]. Z-values represent standard deviations from the median across samples for the given cancer type. Log_2_ Spectral count ratio values from CPTAC were first normalized within each sample profile, then normalized across samples. *UCEC* Uterine corpus endometrial carcinoma, *RCC* renal cell carcinoma

**Fig. 3:**
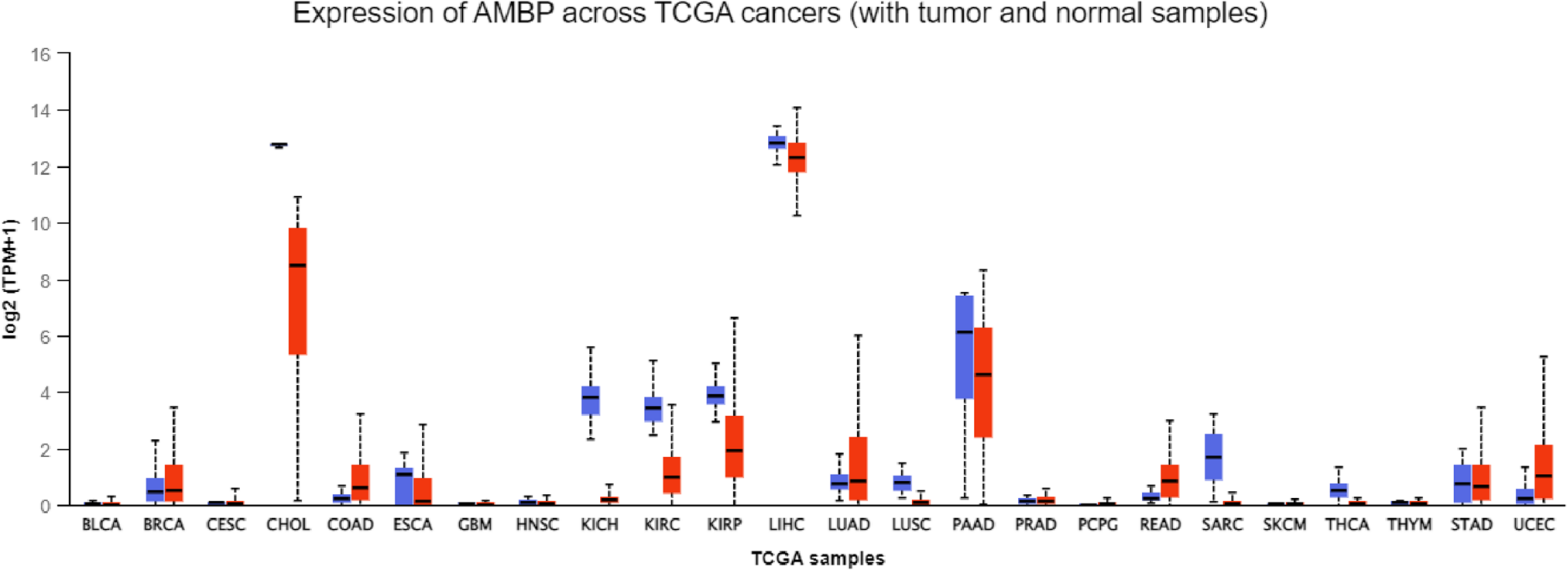
Box-Whisker plot showing AMBP expression across cancers (blue, normal; red, tumor) included in The Cancer Genome Atlas (TCGA) program, obtained by using the web resource UALCAN (http://ualcan.path.uab.edu/cgi-bin/Pan-cancer.pl?genenam=AMBP). *TPM* transcript per million, *BLCA* Bladder urothelial carcinoma; BRCA: Breast invasive carcinoma, *CESC* Cervical squamous cell carcinoma, *CHOL* Cholangiocarcinoma, *COAD* Colon adenocarcinoma, *ESCA* Esophageal carcinoma, *GBM* Glioblastoma multiforme, *HNSC* Head and Neck squamous cell carcinoma, *KICH* Kidney chromophobe, *KIRC* Kidney renal clear cell carcinoma, *KIRP* Kidney renal papillary cell carcinoma, *LIHC* Liver hepatocellular carcinoma, *LUAD* Lung adenocarcinoma, *LUSC* Lung squamous cell carcinoma, *PAAD* Pancreatic adenocarcinoma, *PRAD* Prostate adenocarcinoma, *PCPG* Pheochromocytoma and Paraganglioma, *READ* Rectum adenocarcinoma, *SARC* Sarcoma, *SKCM* Skin cutaneous melanoma, *THCA* Thyroid carcinoma, *THYM* Thymoma, *STAD* Stomach adenocarcinoma, *UCEC* Uterine corpus endometrial carcinoma

In the last years, the application of the recently developed high-throughput technologies such as next-generation sequencing, microarrays, and mass spectrometry-based proteomics has provided a huge amount of information in many research fields including cancer, frequently freely accessible to the research community. Contextually, several web-tools have been developed to perform in-depth analyses of these data. Among them, the interactive web-portal UALCAN has been proven to be valuable in identifying candidate biomarkers for specific cancers, with diagnostic, prognostic, or therapeutic implications [70, 71]. This resource allows for the analysis of high-throughput gene- and protein-expression data obtained by the CPTAC [72, 73] and TCGA consortium [74]. With the aim of providing new information to the poor literature on the potential of bikunin as tissue and circulating biomarker of cancer, we searched through the most recent cancer proteogenomics databases with the in silico platform UALCAN for evaluating bikunin protein expression (Fig. 2) across tumor and normal samples to be compared with its transcript levels (Fig. 3, Table 1), using AMBP as query gene.

**Table 1:** AMBP expression levels across cancers

		Protein expression*				mRNA**			
		n	Median	Interquartile range	p value***	n	Median	Interquartile range	p value***
Breast cancer	Normal	18	2.087	1.495 − 2.267	< 0.001	114	0.354	0.11 − 0.695	*n.s*
	Tumor	125	0.04	− 0.57 − 0.798		1097	0.346	0.102 − 1.23	
Colon cancer	Normal	100	2.757	2.084 − 3.366	< 0.001	41	0.158	0.095 − 0.255	< 0.001
	Tumor	97	− 0.015	− 0.606 − 0.631		286	0.44	0.136 − 1.466	
Ovarian cancer	Normal	25	2.197	1.566 − 2.653	< 0.001	n.d			
	Tumor	100	− 0.039	− 0.564 − 0.554					
Clear cell renal cell carcinoma	Normal	84	1.775	0.762 − 2.371	< 0.001	72	9.095	6.738 − 12.064	*n.s*
	Tumor	110	0	− 0.639 − 0.682		533	0.896	0.342 − 1.828	
Uterine corpus endometrial carcinoma	Normal	31	2.424	1.95 − 3.263	< 0.001	35	0.195	0.063 − 0.462	< 0.001
	Tumor	100	− 0.045	− 0.53 − 0.43		546	0.804	0.184 − 2.43	
Lung adenocarcinoma	Normal	111	2.452	1.782 − 3.132	< 0.001	59	0.693	0.513 − 1.001	< 0.001
	Tumor	111	− 0.009	− 0.543 − 0.667		515	0.642	0.148 − 2.445	
Pancreatic adenocarcinoma	Normal	74	− 0.722	− 1.273 − − 0.431	< 0.001	4	160.179	80.194 − 170.933	*n.s*
	Tumor	137	0	− 0.56 − 0.823		178	20.558	3.882 − 177.425	
Head and neck squamous carcinoma	Normal	71	2.866	2.403 − 3.637	< 0.001	n.d			
	Tumor	108	− 0.016	− 0.683 − 0.654					
Glioblastoma multiforme	Normal	10	− 1.801	− 1.934 − − 1.753	< 0.001	5	0.04	0.036 − 0.046	*n.s*
	Tumor	99	− 0.018	− 0.693 − 0.585		156	0	0 − 0.035	
Hepatocellular carcinoma	Normal	165	− 0.744	− 1.129 − − 0.358	< 0.001	50	7414.869	6459.472 − 8603.095	< 0.001
	Tumor	165	0	− 0.814 − 0.585		371	4913.81	3358.501 − 7136.313	

*Data obtained from Clinical Proteomic Tumor Analysis Consortium (CPTAC) and the International Cancer Proteogenome Consortium (ICPC) datasets, analyzed with the web resource UALCAN (http://ualcan.path.uab.edu/cgi-bin/Pan-cancer-CPTAC.pl?genenam=AMBP)

**Data for cancers included in The Cancer Genome Atlas (TCGA) program, analyzed with the web resource UALCAN (http://ualcan.path.uab.edu/cgi-bin/Pan-cancer.pl?genenam=AMBP)

***Estimated by Student’s t-test considering unequal variance

*N.s.* not significant, *n.d.* not determined

Protein expression data on 1152 samples across ten types of cancer (breast cancer, colon cancer, ovarian cancer, clear cell renal cell carcinoma, uterine corpus endometrial carcinoma, lung adenocarcinoma, pancreatic adenocarcinoma, head and neck squamous carcinoma, glioblastoma multiforme, hepatocellular carcinoma) and 689 controls were retrieved. Differential analysis showed a significant reduction of protein expression in tumors (p < 0.001) with the only exception of pancreatic adenocarcinoma, glioblastoma multiforme, and hepatocellular carcinoma, where its levels were higher (p < 0.001), compared to controls (Fig. 2). The reduced expression of AMBP in seven of ten cancers investigated, with respect to controls, may further support previous findings suggesting its involvement as anti-cancer and anti-metastatic agent. Although interesting, these analyses present some limitations as they are referred to the AMBP gene. Indeed, as UALCAN does not provide any access to mass spectrometry information, discriminating between bikunin and alpha-1-microglobulin (the other protein product of AMBP translation/processing) expression is not possible.

Transcript expression data on 8609 samples across twenty-four types of cancer and 738 controls were obtained (Fig. 3), evidencing highly significant differences between tumors and controls, estimated by Student’s t-test, for bladder urothelial carcinoma (BLCA), cervical squamous cell carcinoma (CESC), cholangiocarcinoma (CHOL), colon adenocarcinoma (COAD), kidney chromophobe (KICH), liver hepatocellular carcinoma (LIHC), lung adenocarcinoma (LUAD), prostate adenocarcinoma (PRAD), rectum adenocarcinoma (READ), thyroid carcinoma (THCA), stomach adenocarcinoma (STAD), uterine corpus endometrial carcinoma (UCEC) (p < 0.001), and a significant difference for Breast invasive carcinoma (BRCA) (p < 0.05), whereas no differences were found for glioblastoma multiforme (GBM), esophageal carcinoma (ESCA), head and neck squamous cell carcinoma (HNSC), kidney renal clear cell carcinoma (KIRC), kidney renal papillary cell carcinoma (KIRP), lung squamous cell carcinoma (LUSC), pancreatic adenocarcinoma (PAAD), sarcoma (SARC). In respect to pheochromocytoma and paraganglioma (PCPG), skin cutaneous melanoma (SKCM), and thymoma (THYM), statistical tests were not applicable because of the low number of controls.

**Fig. 4:**
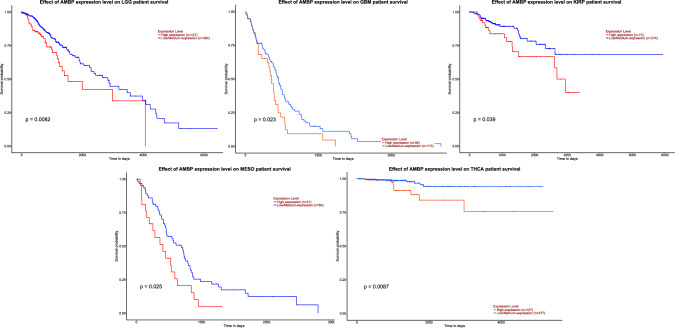
Kaplan–Meier plots showing a significant association between AMBP gene expression and patient’s survival probability in five different cancer types, obtained by the web resource UALCAN. Patients are sorted according to either high (expression value > 3rd quartile) or low AMBP expression levels. Significance is measured by log rank test (p-values < 0.05 are considered to be significant). *LGG* Brain lower grade glioma, *GBM* Glioblastoma multiforme, *KIRP* Kidney renal papillary cell carcinoma, *MESO* Mesothelioma, *THCA* Thyroid carcinoma

The association between AMBP gene expression and patient’s survival in thirty-two different cancer types was also investigated, showing a significant negative impact of high AMBP expression (expression value > 3rd quartile) in brain lower grade glioma (LGG), GBM, KIRP, mesothelioma (MESO), and THCA (Fig. 4). No association was found with regards to the other twenty-seven tumors evaluated (Supplementary Fig. 1).

By comparing protein expression with mRNA levels (whenever possible), widespread discordant patterns were evidenced (Table 1), as also reported in an early work by Sánchez et al. on mouse embryogenesis [21], underling once more that steady-state transcript abundances only partially predict protein levels because of the post-transcriptional, translational, and post-translational regulatory mechanisms occurring after mRNAs are made [75].

## Conclusions

Recent technological advances in mass spectrometry-based proteomics have provided researchers with a huge amount of information allowing for large-scale surveys of the cancer proteome. By using the web-tool UALCAN, we analyzed the freely available proteogenomic data for evaluating bikunin expression across several types of tumors showing common patterns, particularly at the protein level. This represents an interesting starting point that should foster further research focusing on the evaluation of the diagnostic and/or prognostic potential of bikunin, for example by analyzing its levels at different tumor stages, both in tissue and in plasma.

## Supplementary Information

Below is the link to the electronic supplementary material.

Supplementary file1 (PDF 1381 KB)
