# Supplementary material for: Molecular and pathobiological insights of bikunin/UTI in cancer
Source: Mol Biol Rep. 2022 Nov 21;50(2):1701–11. doi: 10.1007/s11033-022-08117-2 (PMC9889512; doi:10.1007/s11033-022-08117-2)
Supplement: Supplementary file 1 — Supplementary file1 (PDF 1381 KB) [file 11033_2022_8117_MOESM1_ESM.pdf]

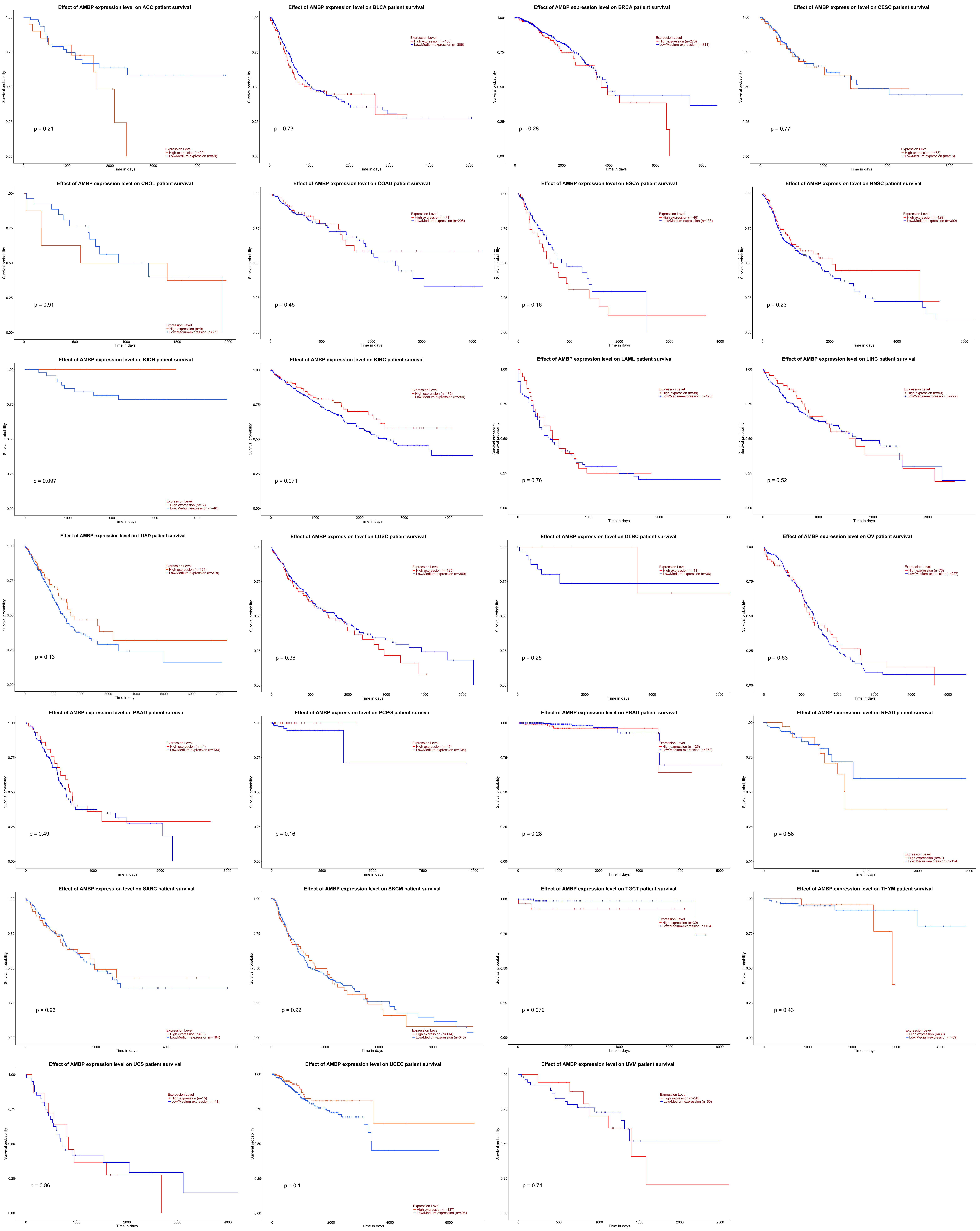


**Supplementary figure 1.** Kaplan-Meier plots showing no effects of AMBP gene expression on patients survival in twenty-seven different cancer types. Patients are sorted according to either high (expression value > 3$^{rd}$ quartile) or low AMBP expression levels. Significance is measured by log rank test (*p-values* <0.05 are considered to be signficant).

ACC: Adrenocortical carcinoma; BLCA: Bladder urothelial carcinoma; BRCA: Breast invasive carcinoma; CESC: Cervical squamous cell carcinoma; CHOL: Cholangiocarcinoma; COAD: Colon adenocarcinoma; ESCA: Esophageal carcinoma; HNSC: Head and Neck squamous cell carcinoma; KICH: Kidney chromophobe; KIRC: Kidney renal clear cell carcinoma; LAML: Acute myeloid leukemia; LIHC: Liver hepatocellular carcinoma; LUAD: Lung adenocarcinoma; LUSC: Lung squamous cell carcinoma; DLBC:Lymphoid Neoplasm Diffuse Large B-cell Lymphoma; OV: Ovarian serous cystadenocarcinoma; PAAD: Pancreatic adenocarcinoma; PCPG: Pheochromocytoma and Paraganglioma; PRAD: Prostate adenocarcinoma; READ: Rectum adenocarcinoma; SARC: Sarcoma; SKCM: Skin cutaneous melanoma; TGCT: Testicular Germ Cell Tumors; THYM: Thymoma; UCS: Uterine Carcinosarcoma; UCEC: Uterine corpus endometrial carcinoma; UVM: Uveal Melanoma.
